# C-reactive protein levels in patients at cardiovascular risk: EURIKA study

**DOI:** 10.1186/1471-2261-14-25

**Published:** 2014-02-24

**Authors:** Julian PJ Halcox, Carine Roy, Florence Tubach, José R Banegas, Jean Dallongeville, Guy De Backer, Eliseo Guallar, Ogün Sazova, Jesús Medina, Joep Perk, Philippe Gabriel Steg, Fernando Rodríguez-Artalejo, Claudio Borghi

**Affiliations:** 1 Wales Heart Research Institute, Cardiff University, Heath Park, Cardiff CF14 4XN, UK; 2 Swansea University College of Medicine, Swansea, UK; 3 INSERM CIE 801 and Epidemiology and Clinical Research Department, Hôpital Bichat-Claude Bernard, Assistance Publique–Hôpitaux de Paris, Paris, France; 4 Université Paris Diderot, Paris, France; 5 Department of Preventive Medicine and Public Health, School of Medicine, Universidad Autónoma de Madrid-IdiPAZ, Madrid, Spain; 6 Centros de Investigación Biomédica en Red (CIBER) of Epidemiology and Public Health (CIBERESP), Madrid, Spain; 7 Inserm U744, Institut Pasteur de Lille, Lille, France; 8 Department of Public Health, University of Ghent, Ghent, Belgium; 9 Departments of Epidemiology and Medicine and Welch Center of Prevention, Epidemiology and Clinical Research, Johns Hopkins Bloomberg School of Public Health, Baltimore, MD, USA; 10 Department of Cardiovascular Epidemiology and Population Genetics, National Center for Cardiovascular Research, Madrid, Spain; 11 AstraZeneca Global Medical Affairs, London, UK; 12 Medical Evidence Centre, Global Medical Affairs, AstraZeneca Farmacéutica Spain, Madrid, Spain; 13 School of Health and Caring Sciences, Linnaeus University, Kalmar, Sweden; 14 Inserm U698, Paris, France; 15 Hôpital Bichat-Claude Bernard, Assistance Publique–Hôpitaux de Paris, Paris, France; 16 Department of Internal Medicine, Ageing and Clinical Nephrology, University of Bologna, Bologna, Italy

**Keywords:** C-reactive protein, Cardiovascular disease, Epidemiology, Risk factors/global assessment

## Abstract

**Background:**

Elevated C-reactive protein (CRP) levels are associated with high cardiovascular risk, and might identify patients who could benefit from more carefully adapted risk factor management. We have assessed the prevalence of elevated CRP levels in patients with one or more traditional cardiovascular risk factors.

**Methods:**

Data were analysed from the European Study on Cardiovascular Risk Prevention and Management in Usual Daily Practice (EURIKA, ClinicalTrials.gov Identifier: NCT00882336), which included patients (aged ≥50 years) from 12 European countries with at least one traditional cardiovascular risk factor but no history of cardiovascular disease. Analysis was also carried out on the subset of patients without diabetes mellitus who were not receiving statin therapy.

**Results:**

In the overall population, CRP levels were positively correlated with body mass index and glycated haemoglobin levels, and were negatively correlated with high-density lipoprotein cholesterol levels. CRP levels were also higher in women, those at higher traditionally estimated cardiovascular risk and those with greater numbers of metabolic syndrome markers. Among patients without diabetes mellitus who were not receiving statin therapy, approximately 30% had CRP levels ≥3 mg/L, and approximately 50% had CRP levels ≥2 mg/L, including those at intermediate levels of traditionally estimated cardiovascular risk.

**Conclusions:**

CRP levels are elevated in a large proportion of patients with at least one cardiovascular risk factor, without diabetes mellitus who are not receiving statin therapy, suggesting a higher level of cardiovascular risk than predicted according to conventional risk estimation systems.

**Trial registration:**

ClinicalTrials.gov Identifier: NCT00882336

## Background

Despite recent improvements in the management of cardiovascular risk factors [1-4], cardiovascular disease (CVD) remains the leading cause of death in Europe [5,6]. Well-established risk factors for CVD include older age, male sex, smoking, elevated cholesterol levels, hypertension and diabetes mellitus [7]. Assessment of these factors can be used to estimate patients’ global cardiovascular risk. Two widely used cardiovascular risk estimation systems are the Systematic Coronary Risk Evaluation (SCORE), developed in Europe to evaluate 10-year risk of cardiovascular mortality [8,9], and the Framingham Risk Score (FRS), developed in the USA to estimate 10-year risk of any cardiovascular event [10].

Global cardiovascular risk estimation is critical for selecting appropriate management options in apparently healthy, asymptomatic individuals according to current clinical guidelines. For example, the European guidelines on CVD prevention recommend prescription of blood pressure medication or lipid-lowering drugs for patients with hypertension or elevated serum cholesterol levels, respectively, but only for those estimated to be at high 10-year cardiovascular risk (cardiovascular mortality of ≥5% according to SCORE [7] or risk of major CVD event of ≥20% according to the FRS). Exceptions are patients who are already known to be at high cardiovascular risk owing to having a history of CVD, diabetes mellitus or chronic kidney disease, or having very high levels of single risk factors [7].

In addition to traditional cardiovascular risk factors, slight elevations of the inflammatory marker C-reactive protein (CRP) may also indicate increased cardiovascular risk [11-13]. Measurement of CRP levels may help to identify patients who are at lower or greater cardiovascular risk than is currently appreciated [13,14]. A recent clinical trial of rosuvastatin in patients who have low levels of low-density lipoprotein cholesterol (LDL-C) (<130 mg/dL [<3.4 mmol/L]) but elevated levels of CRP (≥2.0 mg/L) showed a 44% reduction in cardiovascular event rates in the rosuvastatin treatment group compared with placebo (the Justification for the Use of Statins in Prevention: an Interventional Trial Evaluating Rosuvastatin [JUPITER], ClinicalTrials.gov Identifier: NCT00239681), translating to an absolute risk reduction of 1.2%, and a number needed to treat of 95 for 2 years [15]. The earlier Air Force/Texas Coronary Atherosclerosis Prevention Study (AFCAPS/TexCAPS) had shown very low event rates and no evidence of beneficial effects of statin treatment among lower-risk primary prevention patients who had low levels of both LDL-C and CRP [16]. Elevated CRP levels may thus distinguish a group of patients who are at higher cardiovascular risk than predicted according to conventional risk assessment tools, and who might therefore derive a greater than expected benefit from statin therapy.

The European Study on Cardiovascular Risk Prevention and Management in Usual Daily Practice (EURIKA; ClinicalTrials.gov Identifier: NCT00882336) was recently conducted to assess the management of cardiovascular risk factors in primary care in Europe [17-19]. We have carried out a *post hoc* analysis of this study to assess the prevalence of elevated CRP levels in patients with one or more traditional cardiovascular risk factors, across a range of levels of conventionally estimated global cardiovascular risk. In particular, patients were considered who were estimated to be at intermediate levels of cardiovascular risk according to SCORE and FRS, who did not have diabetes mellitus (a marker of high cardiovascular risk) and who were not already receiving statins (for whom a treatment decision had already been made).

## Methods

### Study design and participants

The EURIKA study (ClinicalTrials.gov Identifier: NCT00882336) was conducted in 12 European countries (Austria, Belgium, France, Germany, Greece, Norway, Russia, Spain, Sweden, Switzerland, Turkey and the UK), selected to represent a broad spectrum of CVD risk across Europe, as well as a variety of different healthcare systems. Data collection started in May 2009 and ended in January 2010, with a 3-month data-collection period for each country. The study protocol was approved by the appropriate clinical research ethics committees in each participating country (lead ethics committee for host institution and sponsor: Comité Ético de Investigación Clínica (CEIC) del Hospital Universitario La Paz [Spain]), and all participating patients provided signed informed consent.

The methods for the study have been reported elsewhere [17]. Briefly, the study sample was selected in a two-stage process that involved the random selection of both physicians and their patients [17,19]. In the first stage, primary care practitioners (PCPs) and specialists involved in CVD prevention (including cardiologists, endocrinologists, and internal medicine specialists) were randomly selected for invitation to participate using the OneKey database (Cegedim Dendrite, Boulogne-Billancourt, France) [20]. In total, 809 physicians (approximately 60 per country) agreed to participate in EURIKA, 64% of whom were PCPs [19]. In the second stage, participating physicians invited patients consecutively visiting the clinic who met the selection criteria (age 50 years or older, free of CVD but having at least one major cardiovascular risk factor; see ‘Patient Characteristics’) [18]. Approximately 600 patients were included per country, with a final population size of 7641. For the present analysis, only patients for whom CRP measurements were available were considered (n = 7565). All patients provided signed informed consent forms. In each country, a random sample of 10% of all study centres underwent a site visit for data monitoring and audit to ensure data quality.

### Patient characteristics

• Age 50 years or older.

• Free of clinical CVD (history of myocardial infarction, stable or unstable angina, stroke or transient ischaemic attack).

• At least one of the following cardiovascular risk factors, assessed from the most recent data in the clinical record, or using anthropometry for obesity.

○ Dyslipidaemia:

▪ LDL-C levels ≥4.1 mmol/L (≥160 mg/dL)

▪ high-density lipoprotein cholesterol (HDL-C) levels <1.036 mmol/L (<40 mg/dL) for men or <1.300 mmol/L (<50 mg/dL) for women

▪ triglyceride levels ≥1.7 mmol/L (≥150 mg/dL)

▪ receiving lipid-lowering medication.

○ Hypertension:

▪ systolic blood pressure (SBP) ≥140 mmHg

▪ diastolic blood pressure (DBP) ≥90 mmHg

▪ receiving antihypertensive medication.

○ Smoking:

▪ current or former smoker, with >100 cigarettes smoked in lifetime.

○ Diabetes mellitus:

▪ fasting plasma glucose ≥7.0 mmol/L (126 mg/dL), or

▪ on antidiabetic medication (insulin or oral medications).

○ Obesity:

▪ body mass index (BMI) ≥30 kg/m^2^, or

▪ waist circumference ≥102 cm in men or ≥88 cm in women.

### Assessment of cardiovascular disease risk factors

Information on participating patients was collected via their medical records, clinical anamnesis, physical examination and a 12-hour fasting blood sample collected within 1 day of the outpatient consultation [17]. Blood samples were sent to a central laboratory in Belgium for analysis (the Bio Analytical Research Corporation NV, Ghent, Belgium). CRP levels were measured by a high-sensitivity immunoturbidimetry method (Roche P-Modular analyzer, Roche, Germany), high-density lipoprotein cholesterol (HDL-C) levels were measured by a modified enzymatic method (Roche P-Modular analyzer, Roche, Germany), total cholesterol levels were measured by the CHOD-PAP method (Roche P-Modular analyzer, Roche, Germany) and triglyceride levels were measured by the GPO-PAP method (Roche P-Modular analyzer, Roche, Germany). LDL-C levels were calculated by the Friedewald formula [21] and glycated haemoglobin (HbA_1c_) levels were measured by ion-exchange chromatography (Menarini 8160, Menarini Diagnostics, Netherlands).

### Metabolic syndrome markers

Metabolic syndrome markers that were considered were: low HDL-C levels (<1.0 mmol/L in men or <1.3 mmol/L in women), high triglyceride levels (≥1.7 mmol/L), high HbA_1c_ levels (≥6%), large waist circumference (cut-off dependent on ethnicity [European Caucasian, Sub-Saharan, Middle East/North African and Afro-American: males ≥94 cm and females ≥80 cm; Asian, South American and Caribbean: males ≥90 cm and females ≥80 cm; Native American: males ≥102 cm and females ≥88 cm], in line with clinical guidelines [22]) and high blood pressure (systolic blood pressure ≥130 mmHg and/or diastolic blood pressure ≥85 mmHg, or taking antihypertensive medication). Global cardiovascular risk was estimated according to the SCORE and FRS systems [8,10].

### Statistical analysis

Statistical analyses were carried out using SAS (V9.2, SAS Institute Inc., Cary, NC, USA). Associations between demographic factors and log CRP values were assessed using Pearson correlation coefficients for continuous variables, analysis of variance for categorical values in three or more categories, and Student’s *t*-tests for binary data. A multivariate linear regression model was developed, adjusted for all factors associated with log CRP values in univariate analysis. Statistical significance was defined as two-sided *P* < 0.05.

## Results

### Demographic and baseline characteristics

The demographic and baseline characteristics for the overall study population, the subgroup of patients without diabetes mellitus who were not receiving statin therapy, and the subgroup of those estimated to be at an intermediate risk (SCORE: 10-year risk of death ≥1% to <5% without diabetes mellitus) who were not receiving statin therapy are shown in Table 1.

**Table 1:** Baseline characteristics in the overall population, and in patients without diabetes mellitus and not receiving statins

	**Overall**	**Patients without diabetes mellitus who were not receiving statin treatment**	**Patients at intermediate risk**^**a**^**and not receiving statin treatment**
	**(N = 7565)**	**(n = 3434)**	**(n = 1573)**
Age, years	63.2 (9.0)	61.9 (9.2)	58.2 (5.6)
Women, n (%)	3903 (51.6)	1872 (54.5)	909 (57.8)
Dyslipidaemia, n (%)	4372 (57.8)	879 (25.6)	439 (27.9)
Total cholesterol, mmol/L	5.5 (1.1)	5.8 (1.0)	5.8 (1.0)
LDL-C, mmol/L	3.2 (1.0)	3.6 (0.9)	3.6 (0.9)
HDL-C, mmol/L	1.4 (0.4)	1.5 (0.4)	1.5 (0.4)
Triglycerides, mmol/L	1.8 (1.3)	1.7 (1.2)	1.7 (1.0)
Hypertension, n (%)	5496 (72.7)	2471 (72.0)	1076 (68.4)
SBP, mmHg	135.0 (16.6)	135.5 (16.8)	132.4 (14.6)
DBP, mmHg	80.9 (9.9)	82.1 (10.1)	82.0 (9.6)
Diabetes mellitus, n (%)	2027 (26.8)	0 (0.0)	0 (0.0)
BMI, kg/m^2^	28.9 (5.4)	28.4 (5.4)	28.8 (5.5)
Obese, n (%)	3288 (43.5)	1357 (39.5)	677 (43.0)
Current smoker, n (%)^b^	1594 (21.3)	862 (25.5)	423 (26.9)
CRP, mg/L	4.2 (8.7)	4.3 (8.4)	4.2 (7.5)

Data are mean (standard deviation) unless otherwise indicated.

^a^Intermediate risk defined as a SCORE estimate of ≥1% to <5%.

^b^Percentages were calculated for the total number of patients for whom data were available.

Overall, the mean age of patients was 63.2 years, and approximately 50% were women. Almost 60% of patients had dyslipidaemia, more than 70% had hypertension, just over 25% had diabetes mellitus and more than 40% were obese (mean body mass index [BMI] was approximately 30 kg/m^2^). The characteristics of patients in the subgroup without diabetes mellitus who were not receiving statin therapy were similar to those of the overall population, except for a lower proportion of patients with dyslipidaemia. Mean levels of total cholesterol, LDL-C, HDL-C and triglycerides in the overall population and in patients without diabetes mellitus who were not receiving statin treatment were similar. The characteristics of patients in the subgroup of those at intermediate risk who were not receiving statin therapy were also largely similar to those of the overall population, except for a lower proportion of individuals with dyslipidaemia.

### Factors correlating with CRP levels

**Figure 1:**
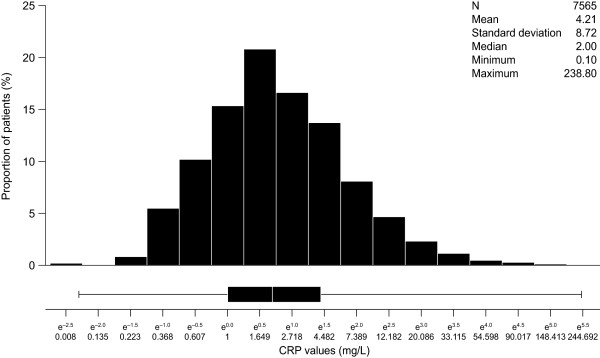
**Distribution of CRP levels in the overall population.** The x axis is a logarithmic scale, with absolute values indicated. The box plot underneath the graph indicates minimum, maximum, median and interquartile range.

The mean CRP level in the overall population was 4.2 mg/L, and this was similar to levels seen in the subgroup of patients without diabetes mellitus who were not receiving statin therapy, and in the subgroup of those at intermediate risk and not receiving statin therapy (Table 1). The distribution of log CRP levels in the overall patient population is shown in Figure 1. The distribution of CRP values is skewed to the right; hence, associations between demographic factors and CRP levels were assessed using the logarithm of CRP values. After univariate analysis, a multivariate linear regression model was developed, adjusted for the values of all factors found to be associated with log CRP levels in univariate analysis. In multivariate analysis, CRP levels were significantly (*P* < 0.05) positively associated with BMI and HbA_1c_ levels (although these associations were of a small magnitude), and were negatively associated with HDL-C levels (Table 2). CRP levels were also significantly higher in: women than in men; patients at an estimated high global cardiovascular risk than in those at an estimated low global cardiovascular risk; and those with several metabolic syndrome markers than in those with no metabolic syndrome markers. No significant association was found between CRP levels and age, although the study included only individuals aged over 50 years.

**Table 2:** Association of log-CRP levels with demographic and clinical characteristics: multivariate analysis in the overall population

	**Linear regression coefficient (SE)**	***P*** **value**
Sex		
Male	–	–
Female	0.23 (0.03)	<0.0001
Cardiovascular risk		
Low^a^	–	–
Intermediate^b^	0.16 (0.05)	0.0005
High^c^	0.21 (0.05)	<0.0001
HDL-C, mmol/L	-0.24 (0.04)	<0.0001
BMI, kg/m^2^	0.05 (0.00)	<0.0001
HbA_1c_,%	0.06 (0.01)	<0.0001
Number of metabolic syndrome markers present		
0	–	–
1	0.02 (0.08)	0.8226
2	0.07 (0.08)	0.3584
3	0.14 (0.08)	0.0807
4	0.20 (0.09)	0.0197
5	0.16 (0.10)	0.0992

Values were adjusted for factors found to be associated with log CRP levels in univariate analysis, including country, sex, systolic blood pressure, HDL-C levels, BMI, HbA_1c_, number of metabolic syndrome markers and cardiovascular risk category.

^a^SCORE <1%, without diabetes mellitus.

^b^SCORE ≥1% to <5%, without diabetes mellitus.

^c^SCORE ≥5%, or with diabetes mellitus.

**Figure 2:**
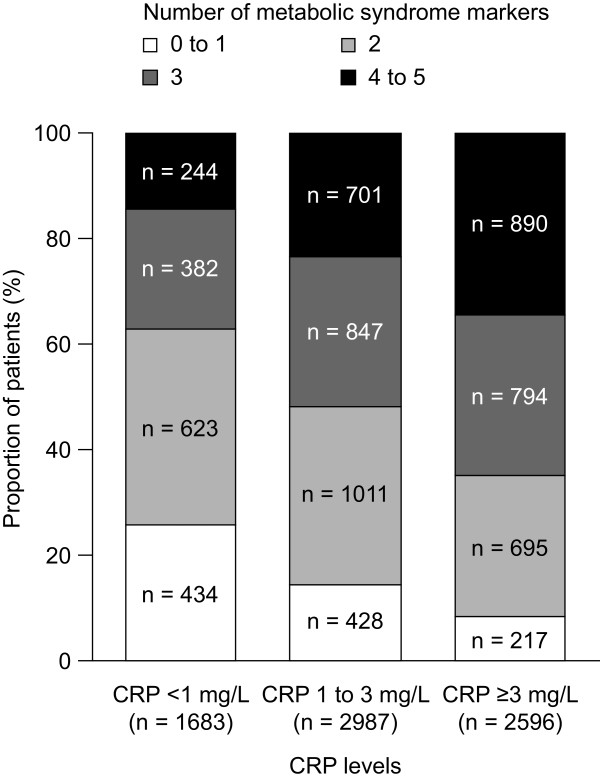
Number of metabolic syndrome components present in patients with different CRP levels.

In the overall population, patients with higher levels of CRP were more likely to have a greater number of metabolic syndrome markers than those with lower levels of CRP (Figure 2). A total of 34.3% of patients with CRP levels ≥3 mg/L had 4 or 5 metabolic syndrome markers, compared with 14.5% of patients with CRP levels <1 mg/L. Furthermore, only 8.4% of patients with CRP levels ≥3 mg/L had 0 or 1 metabolic syndrome markers, compared with 25.8% of patients with CRP levels <1 mg/L. This trend was also apparent when CRP levels were considered in categories of <2 mg/L and ≥2 mg/L (Additional file 1: Figure S1). Similarly, CRP levels were progressively higher in patients with greater numbers of metabolic syndrome markers (Additional file 2: Figure S2). The proportion of patients in the overall population and with CRP levels <1 mg/L, 1–3 mg/L and ≥3 mg/L with each metabolic syndrome marker are shown in Table 3. The prevalence of each metabolic syndrome marker increases with increasing CRP levels.

**Table 3:** Metabolic syndrome markers, overall and according to CRP levels

	**Overall**	**CRP <1 mg/L**	**CRP 1 to 3 mg/L**	**CRP ≥3 mg/L**
	**(N = 7565)**	**(n = 1739)**	**(n = 3104)**	**(n = 2722)**
	**n (%)**	**n (%)**	**n (%)**	**n (%)**
Low HDL-C^a^	1690 (22.3)	240 (13.8)	612 (19.7)	838 (30.8)
High triglycerides^b^	3046 (40.3)	514 (29.6)	1237 (39.9)	1295 (47.6)
High HbA_1c_^c^	2781 (36.8)	493 (28.3)	1092 (35.2)	1196 (43.9)
Large waist circumference^d^	6150 (81.3)	1223 (70.3)	2571 (82.8)	2356 (86.6)
High blood pressure^e^	6436 (85.1)	1390 (79.9)	2628 (84.7)	2418 (88.8)

^a^<1.0 mmol/L in men or <1.3 mmol/L in women.

^b^≥1.7 mmol/L.

^c^≥6%.

^d^Cut-off dependent on ethnicity (European Caucasian, Sub-Saharan, Middle East/North African and Afro-American: males ≥94 cm and females ≥80 cm; Asian, South American and Caribbean: males ≥90 cm and females ≥80 cm; Native American: males ≥102 cm and females ≥88 cm), in line with clinical guidelines [22]).

^e^Systolic blood pressure ≥130 mmHg and/or diastolic blood pressure ≥85 mmHg, or taking antihypertensive medication.

### Elevated CRP levels according to global cardiovascular risk category

**Figure 3:**
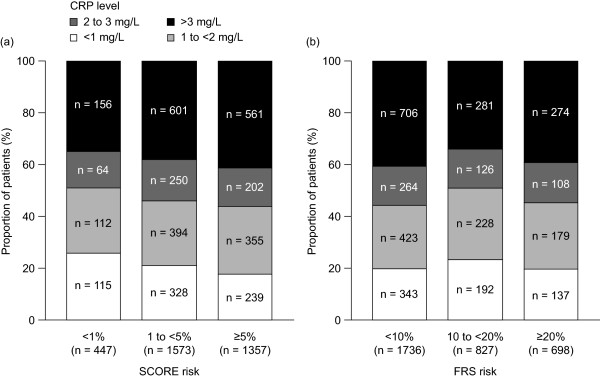
CRP levels according to predicted cardiovascular risk by (a) SCORE and (b) FRS, in patients without diabetes mellitus who were not receiving statin treatment.

Of patients without diabetes mellitus who were not receiving statin treatment, more than one-third (range: 34.0 to 41.3%) of patients in each risk category, whether classified according to either SCORE or FRS, had CRP levels ≥3 mg/L (Figure 3). Approximately 40% of all patients had CRP levels in the range of 1 to 3 mg/L, and approximately 20% had CRP levels <1 mg/L. The proportion of patients with CRP levels <2 mg/L and ≥2 mg/L was also assessed (Additional file 3: Figure S3). Approximately half of all patients had CRP levels ≥2 mg/L, regardless of estimated global cardiovascular risk. Specifically, among those at an estimated intermediate (1 to <5%) 10-year risk of cardiovascular death according to SCORE, 38.2% had CRP levels ≥3 mg/L, and 54.1% had CRP levels ≥2 mg/L. Among those at an estimated intermediate (10 to 20%) 10-year risk of any cardiovascular event according to FRS, 34.0% had CRP levels ≥3 mg/L and 49.2% had CRP levels ≥2 mg/L.

## Discussion

In this analysis of data from a large, multinational European study of the control of cardiovascular risk factors, we have shown that, among patients aged 50 years or older with at least one traditional cardiovascular risk factor who do not have diabetes mellitus and are not receiving statin treatment, more than one-third have CRP levels ≥3 mg/L, and approximately half have CRP levels ≥2 mg/L. This is the case irrespective of the global cardiovascular risk score, estimated using the two most widely used conventional risk estimation systems (SCORE and FRS). In particular, 34.0 to 38.2% of patients without diabetes mellitus who were not receiving statin therapy, and at an estimated intermediate 10-year cardiovascular risk according to SCORE or FRS, had CRP levels ≥3 mg/L, and 49.2 to 54.1% had CRP levels ≥2 mg/L. Elevated baseline CRP levels are associated with increased cardiovascular risk [11,12]; hence, it may be more appropriate to consider classifying such patients in the high-risk category, for whom pharmaceutical intervention for risk factor management may be appropriate [7,23]. The high prevalence of elevated CRP levels in patients considered to be at intermediate risk indicates that assessment of CRP levels in these individuals is likely to identify a considerable number of patients who are at higher risk than would be expected on the basis of their conventional risk factors alone. Patients with diabetes mellitus were excluded from the analysis because they were already considered to be at high risk [7], and patients receiving statins were excluded because a positive treatment decision had already been made. Thus, almost a quarter (23.3%) of the EURIKA study patients without diabetes mellitus and not already taking a statin were found to be at intermediate risk by SCORE and had CRP levels ≥2 mg/L (17% had CRP levels ≥3 mg/L). These patients represent approximately 10% (and 8% respectively) of the entire EURIKA study cohort.

Data from the JUPITER study support the hypothesis that measurement of CRP levels could be used to identify individuals who are likely to benefit from statin therapy, although this approach is not currently endorsed by European guidelines. In this study, ‘high-risk’ cardiovascular event rates were observed among patients receiving placebo treatment with CRP levels ≥2 mg/L, but considered to be at intermediate cardiovascular risk according to SCORE and FRS [15]. Statin treatment in this population resulted in a greater absolute risk reduction than would have been predicted on the basis of reductions in levels of LDL-C alone [15,24]. These data from the EURIKA study show that such individuals are very common in primary care in Europe. We have also shown that, in the overall population, CRP levels are positively associated with BMI, HbA_1c_ levels and the number of components of the metabolic syndrome present, and are negatively associated with HDL-C levels. We observed that CRP levels were higher in women than in men, and higher in those predicted to be at high cardiovascular risk than in those predicted to be at low cardiovascular risk according to SCORE.

Although there is considerable and consistent evidence for an association between CRP levels and cardiovascular risk, the use of CRP measurement in cardiovascular risk assessment remains controversial [25,26]. A meta-analysis published in 2004 of 22 population-based prospective studies, including a total of 7068 incident cases of coronary heart disease, found an adjusted odds ratio for the incidence of coronary heart disease of 1.6 (95% confidence interval 1.5 to 1.7) in patients with baseline CRP levels in the top third of the population analysed (approximately ≥2.4 mg/L), compared with patients with baseline CRP levels in the bottom third (approximately 1.0 mg/L) [11]. However, as our data support, CRP levels are positively associated with several established cardiovascular risk factors, including high blood pressure, atherogenic dyslipidaemia, high BMI, diabetes mellitus, metabolic syndrome and smoking, and are low in individuals with protective factors, including high levels of physical activity, high HDL-C and apolipoprotein A1 levels, and consumption of fruits and vegetables [27-32]. Incomplete adjustment for confounding factors in multivariate analysis may therefore lead to an overestimation of the strength of association between CRP levels and risk. In support of this, as the number of established cardiovascular risk factors adjusted for in multivariate analysis models increases, the correlation of CRP levels with cardiovascular risk decreases [33]. Although our multivariate analysis model was adjusted for all factors found to be associated with CRP levels in univariate analysis, it is possible that the results could still be confounded by CVD risk factors that were not assessed and which are associated with CRP levels, such as dietary habits, levels of physical activity, and low-grade infection such as periodontal disease. The weak independence of CRP levels as a predictor of cardiovascular risk makes it unlikely that increased CRP is a major causal factor for CVD. Moreover, there is no association between cardiovascular event rates and genetic factors that raise CRP levels but have no effect on other cardiovascular risk factors [33]. Such an association would be expected if elevated CRP levels are genuinely causally linked to CVD.

Additionally, a narrative review published in 2006 questioned the value of the universal use of CRP measurements in global cardiovascular risk estimation, on the basis that a very high adjusted odds ratio would be required to provide a moderate improvement in the accuracy of current risk-estimation systems [25]. However, this review did acknowledge that elevated CRP levels may help to distinguish patients who are currently classified as being at intermediate risk using conventional estimation systems, but who may have event rates comparable to those in high estimated risk groups [25,34]. It should also be noted that CRP levels differ considerably between individuals and may fluctuate over time within individuals, so developing a universal system for assessing cardiovascular risk based on single measurements and cut-off CRP values may be challenging [35-40]. However, their variability appears no greater than that of blood pressure and lipid levels. After the initial measurement of CRP levels, repeat measurements can be made at follow-up visits to the PCP.

JUPITER reported that patients with low levels of LDL-C but high levels of CRP benefitted from statin therapy, while the earlier AFCAPS/TexCAPS study found that patients with low levels of both LDL-C and CRP did not [15,16]. In contrast to both of these findings, an analysis of the Heart Protection Study showed that patients benefitted from statin therapy regardless of either their LDL-C or CRP levels [41]. Furthermore, an analysis of data from the Anglo-Scandinavian Cardiac Outcomes Trial (ASCOT) showed that CRP levels did not predict the efficacy of statin treatment in patients with LDL-C levels ≤250 mg/dL (6.5 mmol/L) [42]. However, the majority of patients recruited into these studies would already be considered to be at high cardiovascular risk on the basis of established CVD, diabetes mellitus, or severe or complicated hypertension. These studies are therefore less relevant to the main question considered here, specifically regarding how we might improve the identification of individuals from lower-risk groups who might derive greater than otherwise expected reductions in their absolute CVD risk with more intensive risk factor treatment. It should be noted that the AFCAPS/TexCAPS study used lovastatin, a less potent agent than rosuvastatin, which was used in the JUPITER study [15,16]. It is therefore possible that the intensity of treatment in the AFCAPS/TexCAPS study was not sufficient for an outcome benefit to be detected in the lower-risk subgroup of patients with low levels of both LDL-C and CRP.

Our study has the strength of centralized assessment of a large sample of patients from multiple countries according to standardized procedures. The participation acceptance rate among physicians was low (3.1 to 22.8% across all countries), but the random selection of patients and a relatively high patient acceptance rate of 62.1% within the participating physicians’ practices is likely to have reduced patient selection bias. It should also be noted that, because the data-collection period for each country was only 3 months, it is possible that frequent healthcare service users were over-represented in the study cohort. This may bias the patient population towards the inclusion of less healthy patients, who could have higher CRP levels than healthier individuals.

## Conclusions

In conclusion, we have shown that a large proportion of patients aged over 50 years and with at least one traditional cardiovascular risk factor but no previous history of CVD, who do not have diabetes mellitus and who are not currently being treated with statins, have elevated levels of CRP. This is true regardless of these patients’ levels of cardiovascular risk, estimated according to conventional risk-estimation systems. This is of particular importance for patients currently classified as being at intermediate risk, for whom elevated CRP levels could indicate that they are at higher risk than expected and likely to derive a greater absolute reduction in CVD event rates if pharmacological treatment for risk factor management were provided [7]. For these patients, evaluation of CRP levels is therefore likely to offer a meaningful clinical benefit.

## Abbreviations

BMI: Body mass index; CRP: C-reactive protein; CVD: Cardiovascular disease; EURIKA: EUropean study on cardiovascular RIsK prevention and mAnagement in usual daily practice; FRS: Framingham Risk Score; HbA1c: Glycated haemoglobin; HDL-C: High-density lipoprotein cholesterol; IQR: Interquartile range; JUPITER: Justification for the Use of Statins in Prevention: an Interventional Trial Evaluating Rosuvastatin; LDL-C: Low-density lipoprotein cholesterol; PCP: Primary care physician; SCORE: Systematic COronary Risk Evaluation; SE: Standard error.

## Competing interests

JPJH and JD have received speaker and consulting fees from AstraZeneca; FT has received research funding from AstraZeneca; PGS has received research grants from Sanofi, Servier and the New York University School of Medicine, speaking or consulting fees from Amarin, Astellas, AstraZeneca, Bayer, Boehringer Ingelheim, Bristol-Myers Squibb, Daiichi Sankyo-Lilly, GlaxoSmithKline, Iroko, Medtronic, MSD, Novartis, Otsuka, Pfizer, Roche, Sanofi, Servier and The Medicines Company, and is a stockholder and cofounder of Aterovax. JM and OS are employees of AstraZeneca. The rest of the authors declare that they have no competing interests.

## Authors’ contributions

All authors contributed to the design and conduct of the study, and to the drafting and final approval of the manuscript.

## Pre-publication history

The pre-publication history for this paper can be accessed here:

http://www.biomedcentral.com/1471-2261/14/25/prepub

## Supplementary Material

Additional file 1: Figure S1 Number of metabolic syndrome components present in patients with CRP levels <2 mg/L and ≥2 mg/L. Click here for file

Additional file 2: Figure S2 Mean (IQR) of CRP levels in patients with different numbers of metabolic syndrome components. Click here for file

Additional file 3: Figure S3 CRP levels as a two-category variable (<2 mg/L and ≥2 mg/L) according to predicted cardiovascular risk levels by (a) SCORE and (b) FRS, in patients without diabetes mellitus who were not receiving statin treatment. Click here for file
