# Supplementary material for: C-reactive protein levels in patients at cardiovascular risk: EURIKA study
Source: BMC Cardiovasc Disord. 2014 Feb 24;14:25. doi: 10.1186/1471-2261-14-25 (PMC3943833; doi:10.1186/1471-2261-14-25)
Supplement: Additional file 1: Figure S1 — Number of metabolic syndrome components present in patients with CRP levels <2 mg/L and ≥2 mg/L. [file 1471-2261-14-25-S1.pdf]

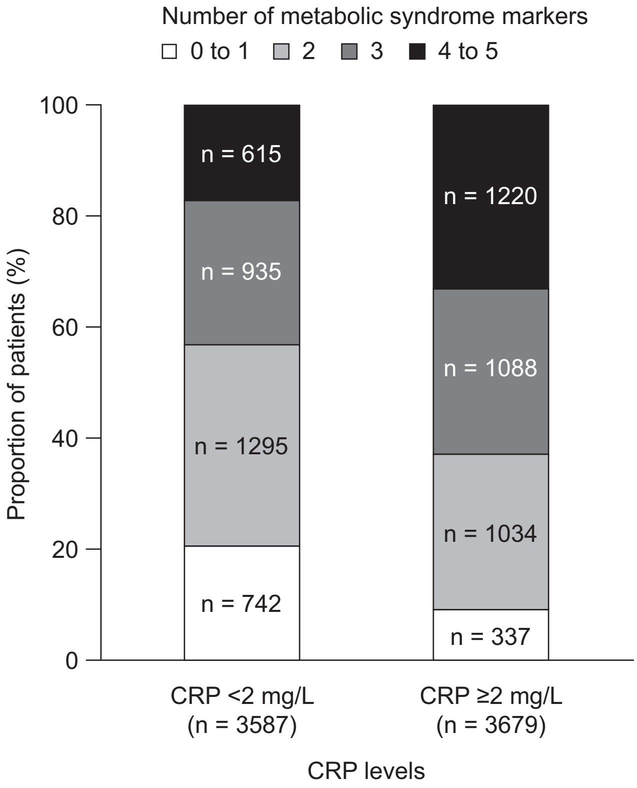

Additional file 1: Figure S1. Number of metabolic syndrome components present in patients with CRP levels <2 mg/L and ≥2 mg/L.

Halcox *et al.*: **C-reactive protein levels in patients at cardiovascular risk: EURIKA study.** *BMC Cardiovascular Disorders* 2014 **14**:25.
