# Supplementary material for: C-reactive protein levels in patients at cardiovascular risk: EURIKA study
Source: BMC Cardiovasc Disord. 2014 Feb 24;14:25. doi: 10.1186/1471-2261-14-25 (PMC3943833; doi:10.1186/1471-2261-14-25)
Supplement: Additional file 2: Figure S2 — Mean (IQR) of CRP levels in patients with different numbers of metabolic syndrome components. [file 1471-2261-14-25-S2.pdf]

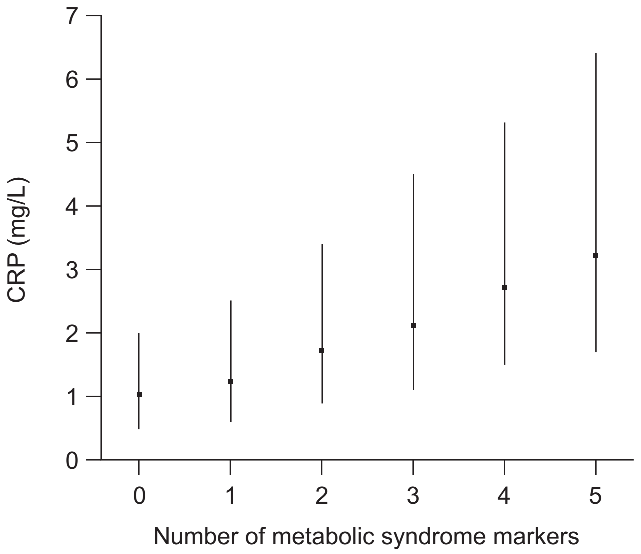

Additional file 2: Figure S2. Mean (IQR) of CRP levels in patients with different numbers of metabolic syndrome components.

Halcox *et al.*: **C-reactive protein levels in patients at cardiovascular risk: EURIKA study.** *BMC Cardiovascular Disorders* 2014 **14**:25.
