# Supplementary material for: C-reactive protein levels in patients at cardiovascular risk: EURIKA study
Source: BMC Cardiovasc Disord. 2014 Feb 24;14:25. doi: 10.1186/1471-2261-14-25 (PMC3943833; doi:10.1186/1471-2261-14-25)
Supplement: Additional file 3: Figure S3 — CRP levels as a two-category variable (<2 mg/L and ≥2 mg/L) according to predicted cardiovascular risk levels by (a) SCORE and (b) FRS, in patients without diabetes mellitus who were not receiving statin treatment. [file 1471-2261-14-25-S3.pdf]

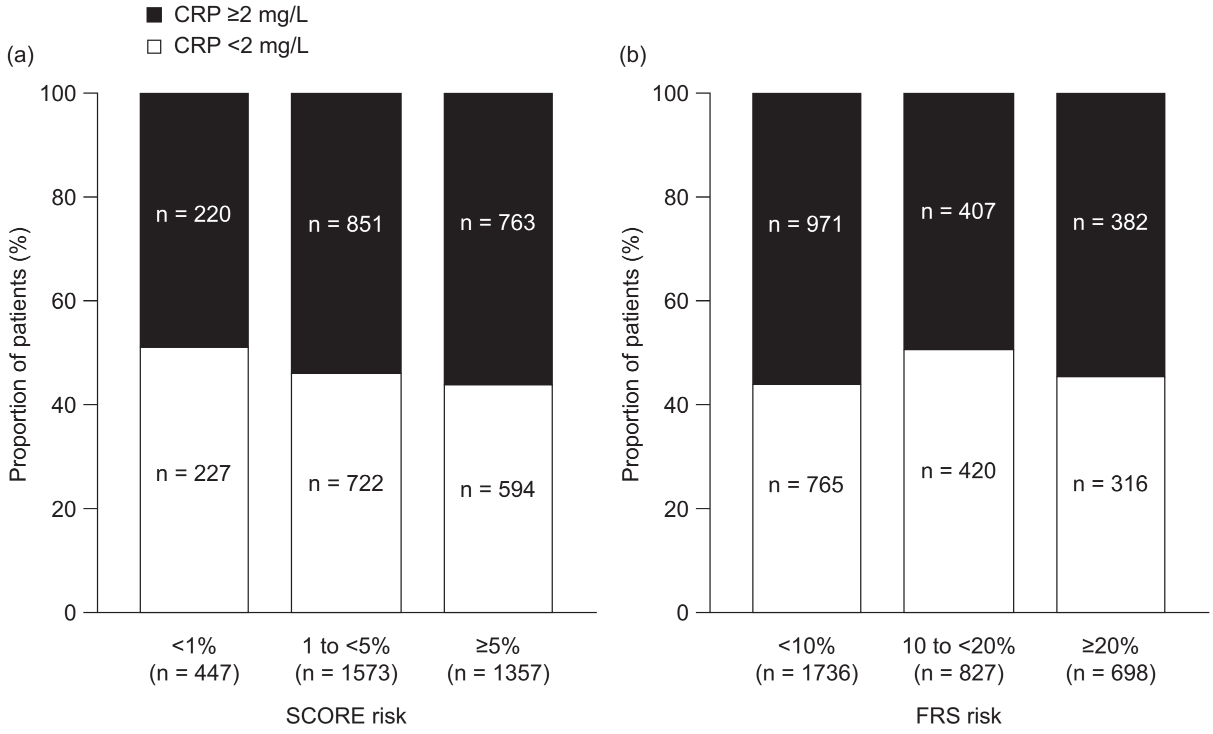

Additional file 3: Figure S3. CRP levels as a two-category variable (<2 mg/L and ≥2 mg/L) according to predicted cardiovascular risk levels by (a) SCORE and (b) FRS, in patients without diabetes mellitus who were not receiving statin treatment.

Halcox *et al.*: **C-reactive protein levels in patients at cardiovascular risk: EURIKA study.** *BMC Cardiovascular Disorders* 2014 **14**:25.
